# Melittin as a safe compound to BALB/c mice immune system; a tiered approach immunotoxicity screening

**DOI:** 10.1186/s12906-023-04228-w

**Published:** 2023-10-25

**Authors:** Gholamreza Karimi, Sina Fatemi, Bahram Memar, Mohammad-Bagher Khorrami, Arian Amali, Mahmood Sadeghi, Seyed-Alireza Esmaeili, Bamdad Riahi-Zanjani

**Affiliations:** 1 Pharmaceutical Research Center, Pharmaceutical Technology Institute, Mashhad University of Medical Sciences, Mashhad, Iran; 2 Department of Pharmacodynamics and Toxicology, School of Pharmacy, Mashhad University of Medical Sciences, Mashhad, Iran; 3 School of Pharmacy, Mashhad University of Medical Sciences, Mashhad, Iran; 4 Cancer Research Center, Faculty of Medicine, Mashhad University of Medical Sciences, Mashhad, Iran; 5 Social Security Organization, 17th Shahrivar Hospital, Mashhad, Iran; 6 Student Research Committee, Paramedical Department, Islamic Azad University, Mashhad Branch, Mashhad, Iran; 7 Medical Toxicology and Drug Abuse Research Center (MTDRC), Birjand University of Medical Sciences, Birjand, Iran; 8 Immunology Research Center, Mashhad University of Medical Sciences, Mashhad, Iran; 9 Immunology Department, Faculty of Medicine, Mashhad University of Medical Sciences, Mashhad, Iran; 10 Medical Toxicology Research Center, Mashhad University of Medical Sciences, Mashhad, Iran

**Keywords:** Melittin, Immune system, Humoral immunity, Cellular immunity

## Abstract

**Background:**

Maintenance of immune system integrity is a vital requirement to protect human body against pathogens/cancers. Natural compounds have long been used due to their benefits for the immune system. One of which is bee venom that contains a peptide called melittin having antimicrobial and anticancer effects. Since a limited number of studies regarding the effects of melittin on the immune system have been carried out, we aimed to evaluate the effects of melittin on BALB/c mice immune system parameters.

**Methods:**

Female BALB /c mice were treated intraperitoneally (i.p) with 0.75 and 1.5 mg/kg doses of melittin for 14 days (5 doses per week). The negative control group received i.p normal saline whereas the positive controls received i.p 20 mg/kg cyclophosphamide (CYP). Immunological parameters such as hematological parameters, delayed-type hypersensitivity (DTH), hemagglutination titer (HA), spleen cellularity, splenocytes proliferation, as well as spleen and bone marrow histopathological assessment were evaluated.

**Results:**

Our findings showed that melittin has no gross pathological effect on the spleen and bone marrow. It was also demonstrated that melittin has no any significant effect on hematological parameters. Melittin did not cause any significant changes to proliferation response of splenocytes to PHA and LPS, spleen cellularity, DTH response, as well as the production of anti-SRBC antibodies. According to our results, melittin at 0.75 and 1.5 mg/kg doses could not induce significant changes on immune parameters and as a result, melittin was found to be safe for the mice immune system.

## Introduction

The maintenance of immune system integrity is required for human body to fight pathogens jeopardizing our health, and to prevent the onset of numerous diseases and cancers. Natural compounds have long been a keen interest for their potential immunomodulatory effects due to their acceptable efficacy and minimal side effects [1, 2]. Honey bee venom is a valuable natural compound which is suggested (in studies) as a candidate for the treatment of various ailments such as arthritis, gout, rheumatism, and other disorders related to the immune system [3]. This venom contains antimicrobial peptides which are effective against a broad range of gram-positive/negative bacteria [4]. The efficacy of antimicrobial peptides derived from the venom on septic and non-septic inflammations, wound healing, and regulation of adaptive immune system have also been studied [5].

Melittin a 26-amino acid peptide is the most important active constituent of honey bee venom that forms up to 50% of its dry weight [6], with significant antimicrobial and antiviral properties [7]. In a study conducted in 2005, melittin’s antimicrobial properties on Chlamydia trachomatis and Mycoplasma hominis infections were evaluated, and it was concluded that this substance could act as an agent for the prevention/control of urogenital infections [8]. In another study carried out in 2011, acute pancreatitis was prevented using melittin [9]. Researchers have also tried to alleviate different allergic reactions brought by the use of melittin through altering its molecular structure to reduce its possible side effects [6]. Moreover, melittin has the ability to induce cellular lysis, especially the lysis of the membrane of red blood cells [10]. Its amphiphilic structure provides it to react with and demolish phospholipid bilayer of cell membranes [11]. It has been reported that nanoparticles containing melittin could be effective against HIV, indicating the possibility of utilizing bee venom as a promising treatment for AIDS [12, 13].

Additionally, it has been claimed that melittin has anticancer properties through induction of apoptosis [14], necrosis, and cancer cell lysis [15]; therefore, malignant cells could be targeted [16] by melittin in cancers such as kidney, lung [17], liver [18], and breast cancer [19]. Since melittin has multiple claimed therapeutic features and in other side, there is a limited number of studies regarding its effects on the immune system, we aimed to evaluate the immunotoxic/immunomodulatory properties of melittin in BALB/c mice with a tiered approach immunotoxicity screening.

## Materials and methods

### Animals

Female BALB/c inbred mice were procured from the School of Pharmacy at Mashhad University of Medical Sciences. Animals were housed in polystyrene cages with an ambient temperature of 20–25 ºC under a 12 h light/dark lighting cycle and had no restriction in access to food and water. Animals were allowed to acclimatize for at least one week prior to use. All methods employed for animal experiments were approved by the Ethics committee of Mashhad University of Medical Sciences.

### Chemicals

Phytohemagglutinin-A (PHA), Lypopolysaccharide (LPS), Cyclophosphamide, Melittin, and 3-(4, 5-dimethylthiazol-2-yl)-2, 5-diphenyl tetrazolium bromide (MTT) dye were purchased from Sigma (UK). Fetal bovine serum and RPMI-1640 medium were obtained from Gibco (Spain).

### Doses and exposure schedules

**Fig. 1:**
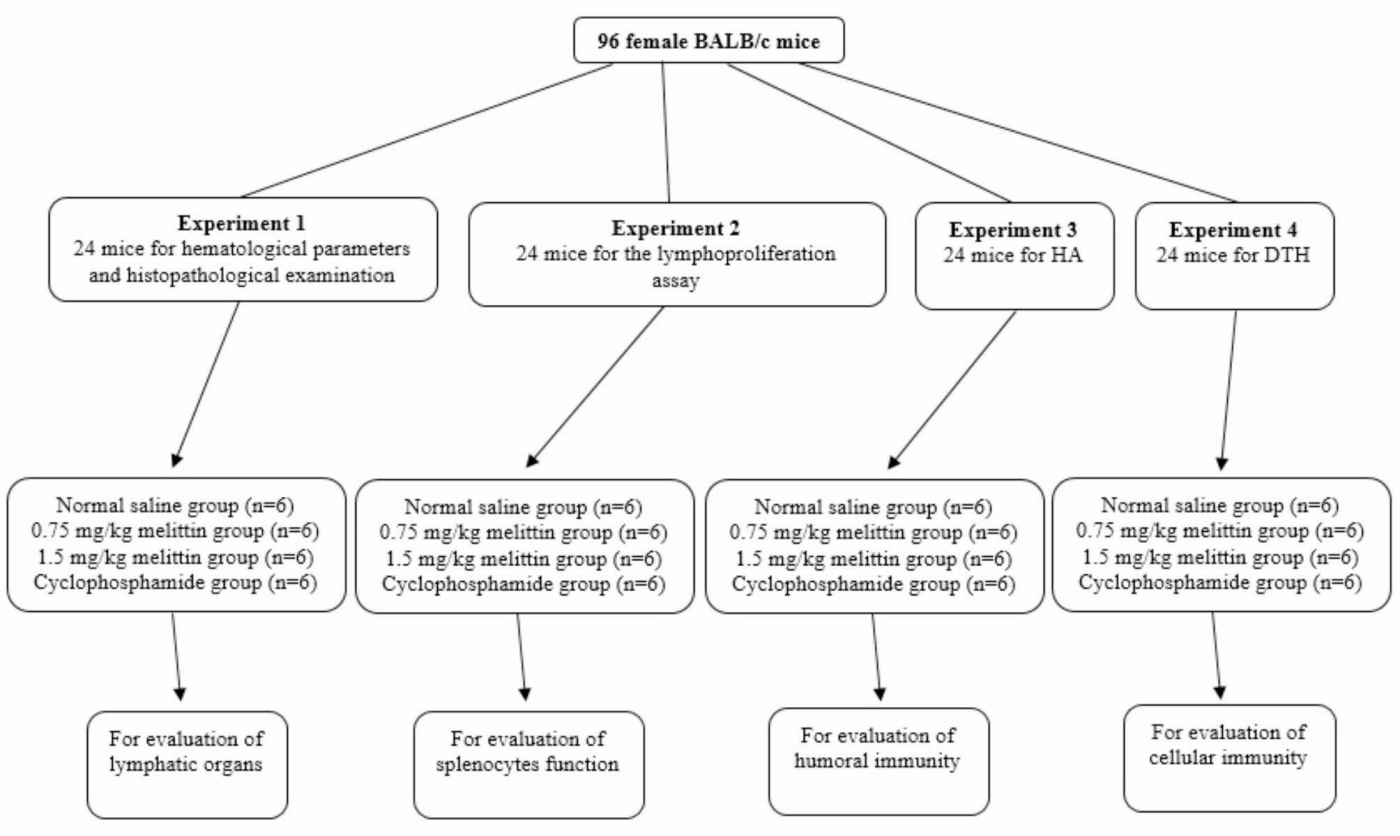
Experimental protocol

Mice were divided into four groups and each group including 24 mice was used for a different set of experiments. First group was used for histopathological examination and hematological parameters. The second group was used for the lymphoproliferation assay. Third group was considered for the evaluation of humoral immunity. Delayed-Type Hypersensitivity (DTH) response in order to evaluate cellular immunity was investigated in forth group of mice. Moreover, the mice in each group were distributed into four subgroups; Subgroup 1 as negative control received normal saline (n = 6); Subgroup 2 received 0.75 mg/kg melittin (n = 6); Subgroup 3 received 1.5 mg/kg melittin (n = 6); and subgroup 4 as positive control received 20 mg/kg cyclophosphamide (n = 6) (Fig. 1). All subgroups were treated ip for 14 days (5 times per week). The criterion for choosing the injected doses of melittin in this study was the amount of determined LD50 of melittin in mice which is 7.4 mg/kg. In this study, 10% and 20% of the aforementioned LD50 (0.75 and 1.5 mg/kg, respectively) were used.

### Histopathological examination

On day 15, mice were anesthetized i.p with 0.2 ml of a ketamine-xylazine (90 mg/kg-10 mg/kg) working solution, and blood samples were drawn from the retro-orbital plexus. Then the animals were sacrificed, and the spleen of each animal was removed and stabilized in a solution of 10% formalin. Subsequently, the process of staining 5-μm-thick sections of these tissues with Hematoxylin & Eosin (H&E) was carried out. The spleen was also evaluated concerning atrophy or white pulp hyperplasia, the ratio of red pulp to white pulp, and deposits in the red and white pulp. In addition, the femurs of each mouse were collected for bone marrow evaluation and after decalcification in 10% Nitric acid, the samples were stained with H&E. Bone marrow smears were examined regarding cellularity, presence and development of the subtypes of hematopoietic cells, and the ratio of erythroid lineage to lymphoid lineage. Histopathological alterations of organs were then investigated using light microscopy [20].

### Determination of hematological parameters

The animals’ blood was used for the evaluation of hematological factors such as white blood cell (WBC) count and differentiation. Two hundred microliters of blood from each mouse was dispensed in a sterile anti-coagulated ethylene diamine tetra acetic acid dipotassium salt (K2-EDTA) tube to allow hematological indexes determinations. Furthermore, a smear of peripheral blood was also provided, stained with Giemsa, and then observed under a light microscope for differential count of leukocytes [20].

### Preparation of single-cell suspension and splenocyte enumeration

Each isolated spleen was placed into a small petri dish containing RPMI-1640 media supplemented with 10% fetal bovine serum (FBS), 100 μg/ml streptomycin, 100 U/ml penicillin, and 2 mM glutathione. Then, a splenic suspension was procured. The suspension was transferred into a falcon tube using a cell strainer with a pore size of 40 μm and centrifuged at 1200 rpm at 4℃ for 10 min. The supernatant was removed and the pellet re-suspended in RBC lysing buffer containing 0.83% NH4Cl in 100 mM Tris buffer, pH 7.4, and kept at room temperature for 3 min. The cells were washed three times with the media and suspended into 1 ml of the media. Using the Neubauer chamber, spleen cellularity was evaluated. The viability of cells was performed using the trypan blue exclusion method [20].

### Lymphocyte proliferation test

The 100 μl aliquots of the splenocytes at 2 × 10^6^ viable cells/ml were dispensed into wells of a 96-well microtiter plate. Afterwards, complete media or Phytohemagglutinin-A (PHA at a final level of 5 μg/ml) or lipopolysaccharide (LPS at a final concentration of 1 μg/ml) was added to triplicate designated wells. The plates were then incubated for 48 h at 37 ºC and 5% CO2 in a humid incubator, and then, using MTT-based assay, cell proliferation was determined. For this purpose, 15 μl of a 5 mg/ml solution of 3- (4, 5-diamethyl-2-thiazolyl) 2, 5-diphenyl-2 H-tetrazolium (MTT); was added to each well and then incubated at 37 ºC in CO2 humid incubator for 4 h. The blue formazan precipitate was then dissolved in dimethylsulfoxide (DMSO), and the optical density in each well was determined at 570 nm by Stat-Fax™ Elisa Reader [20]. The following formula was used to calculate proliferation index (PI):

$${\rm{PI}}\,{\rm{ = }}\,{\rm{Absorbance}}\,{\rm{of}}\,{\rm{stimulated}}\,{\rm{cells/Absorbance}}\,{\rm{of}}\,{\rm{unstimulated}}\,{\rm{cells.}}$$

### Hemagglutination titer assay

On the 9th day of the treatment, the animals were immunized i.p. by 5 × 10^8^ SRBC/100 μl. After 5 days and at the end of the treatment, blood samples were drawn from the retro-orbital plexus of mice and placed in microtubes. Using a centrifuge, the serum of the samples was separated and preserved at -20℃ until testing. The aliquots (50 μl) of two-fold dilutions of the sera (in PBS) were combined with 50 μl of a 2% [v/v] SRBCs suspension in a glass tubes. The tubes were placed at37 ℃ for 2 h, and the intended antibody titer was determined based on the presence of agglutination. The highest dilution giving hemagglutination was considered as the antibody titer [21].

### Delayed-type hypersensitivity response (DTH)

On the 9th day of the treatment, mice were injected/immunized i.p. with 1 × 10^9^ SRBCs. After 5 days (day 14), a booster dose of 1 × 10^8^ SRBCs in the left hind footpad was injected into all animals, and 50 μl of normal saline was injected into the right hind footpad (for evaluation of non-specific swelling) [21]. The increase in the volume of the left footpad was determined after 48 h, and the mean percentage increase in the foot pad thickness was calculated according to the following formula:

$$\left( {{\rm{Left}}\,{\rm{footpad}}\,{\rm{challenged}}\,{\rm{with}}\,{\rm{SRBC}}\,{\rm{ - }}\,{\rm{Right}}\,{\rm{footpad}}} \right)\,{\rm{ \times }}\,{\rm{100/}}\,{\rm{Right}}\,{\rm{footpad}}$$

### Statistical analysis

Data were statistically analyzed by Student’s *t*-test to assess significant changes in the data of different groups. P values less than 0.05 were supposed significant. The values are expressed as means ± SEM.

## Results

### Histopathological examination

#### Bone marrow

**Fig. 2:**
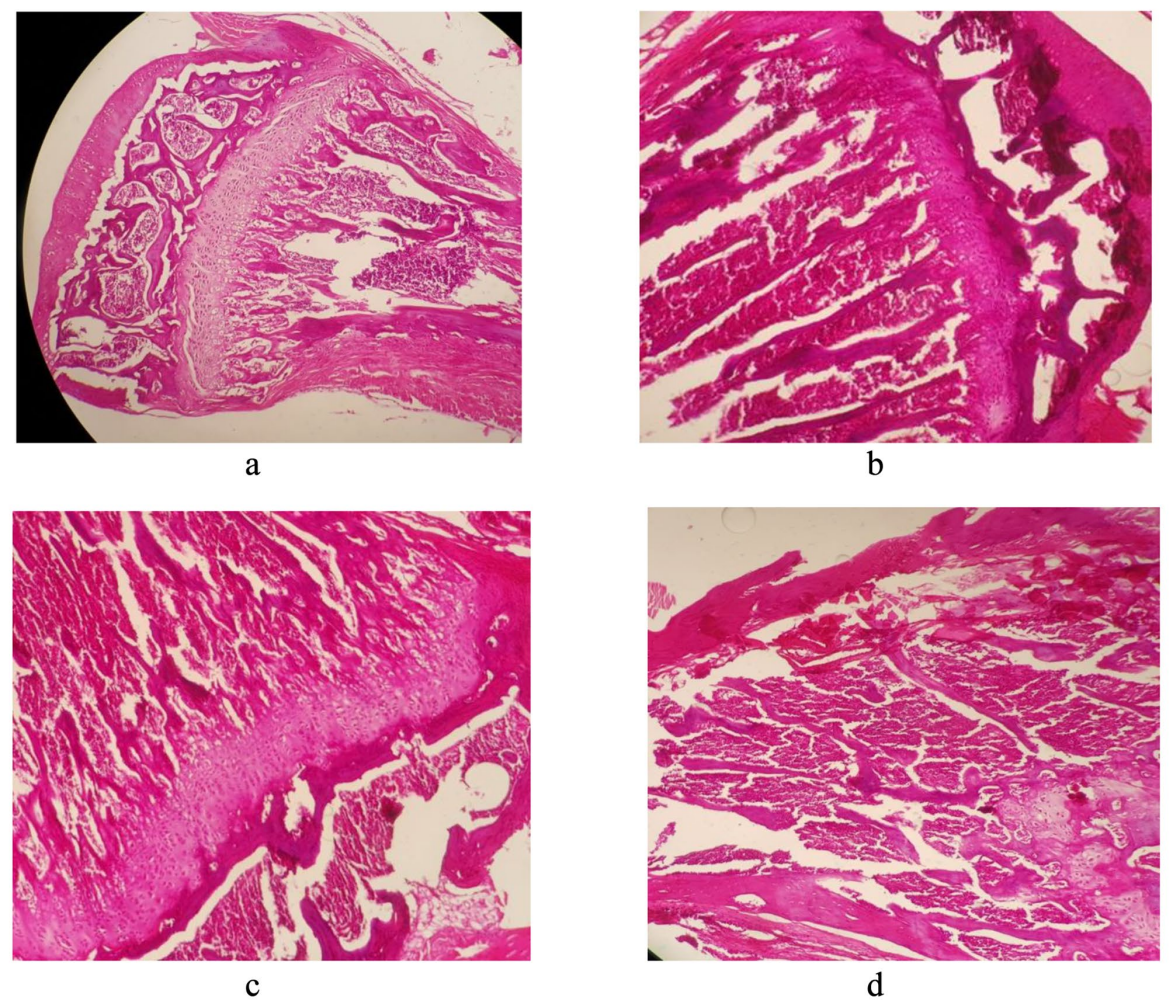
**a**. Bone marrow (normal saline group *100). **b**. Bone marrow (0.75 mg/kg melittin group *100). **c**. Bone marrow (1.5 mg/kg melittin group *100). **d**. Bone marrow (cyclophosphamide group *100)

Some critical indexes such as cellularity, hematopoietic cell subtypes’ presence/maturation, in addition to ratios of the erythroid lineage to myeloid lineage were observationally analyzed. The investigations on different parameters showed that there was not any significant histopathological changes between samples from melittin treated groups relative to normal saline control mice (Fig. 2; a to d).

#### Spleen

**Fig. 3:**
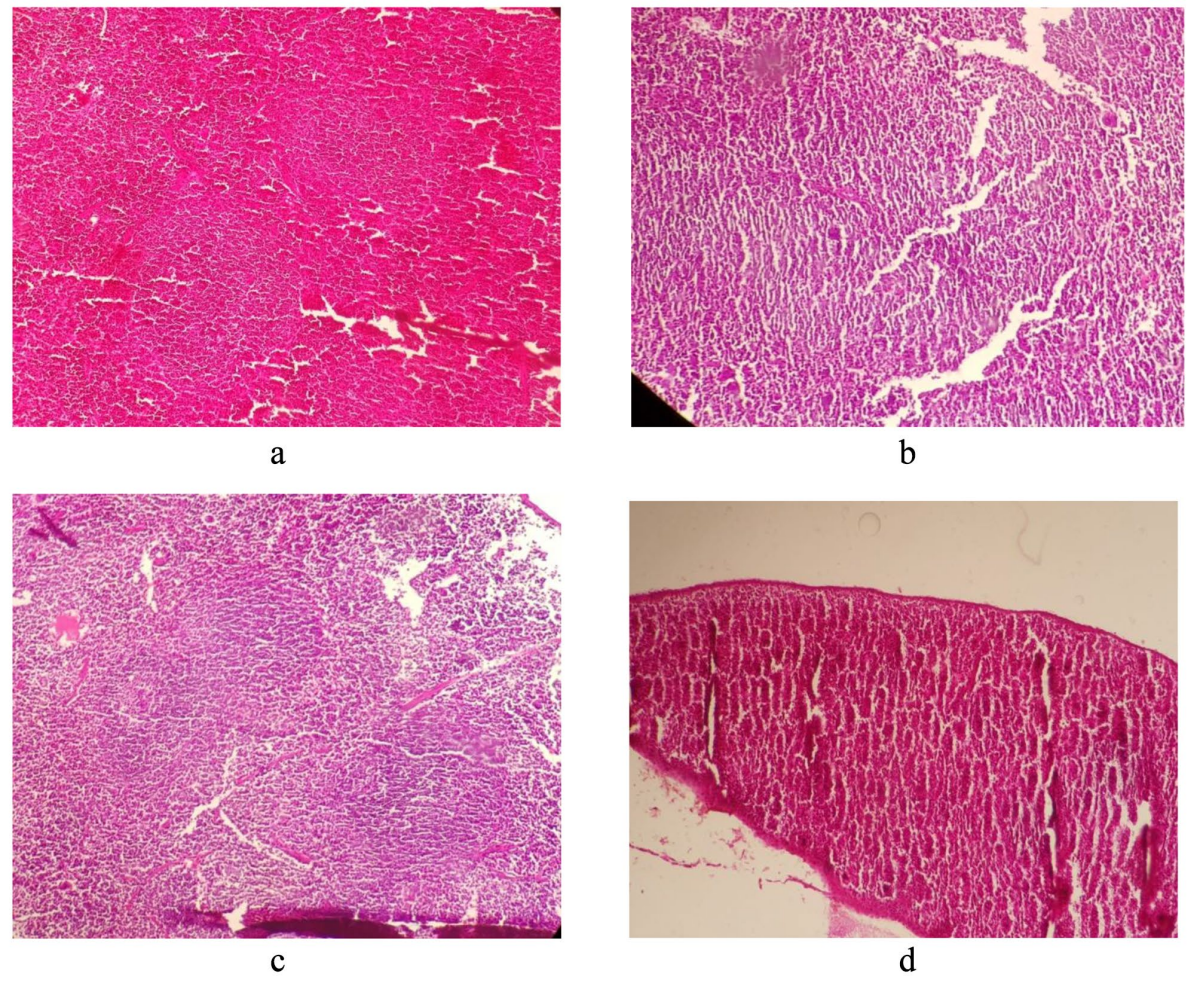
**a**. Spleen (normal saline group *100). **b**. Spleen (0.75 mg/kg melittin group *100). **c**. Spleen (1.5 mg/kg melittin group *100). **d**. Spleen (cyclophosphamide group *100)

Spleen was evaluated with regard to some parameters such as white pulp atrophy/hyperplasia, ratio of white pulp to red pulp, splenic trabecular abnormality, necrosis, apoptosis, clumps, and debris in the white and red pulp regions. The observational evaluation of spleen tissue revealed that melittin at both doses could not cause any significant side effect on spleen when compared to controls (Fig. 3; a to d).

### Hematological parameters

As can be seen in Table 1, both doses of melittin did not induce any significant differences in the studied hematological parameters. On the other hand, in comparison with the negative control group, cyclophosphamide at dose of 20 mg/kg as positive control, caused significant reductions in WBC, lymphocyte (P < 0.001), and neutrophil counts (P < 0.05), as well as spleen cellularity (P < 0.05).

**Table 1:** The subacute effects of i.p administration of melittin on hematological parameters in different groups of mice

Parameters	Normal Saline	Melittin 0.75 mg/kg	Melittin 1.5 mg/kg	CYP 20 mg/kg
White Blood Cells	6783 ± 289	6417 ± 244	5460 ± 554	3900 ± 445^***^
Neutrophil	2137 ± 82	1858 ± 251	1450 ± 397	1505 ± 229^*^
Lymphocyte	4498 ± 301	4395 ± 83	3726 ± 405	2273 ± 259^***^
Monocyte	105 ± 19	100 ± 27	97 ± 31	79 ± 28
Eosinophil	43 ± 14	64 ± 2	32 ± 16	43 ± 17
Spleen cellularity	162 ± 39	123 ± 12	130 ± 26	93 ± 16^*^

Data are shown as mean ± SEM. ^*^P < 0.05, ^***^P < 0.001 indicate significant changes compared to the negative control group

### Lymphocyte proliferation test

**Fig. 4:**
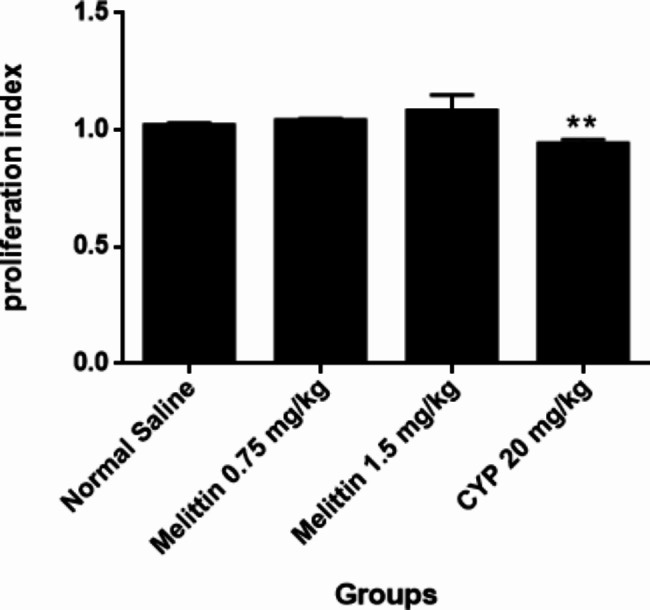
The subacute effects of i.p administration of melittin on PHA response in different groups of mice. Data are shown as mean ± SEM. **P < 0.01 indicates significant changes compared to the negative control group

**Fig. 5:**
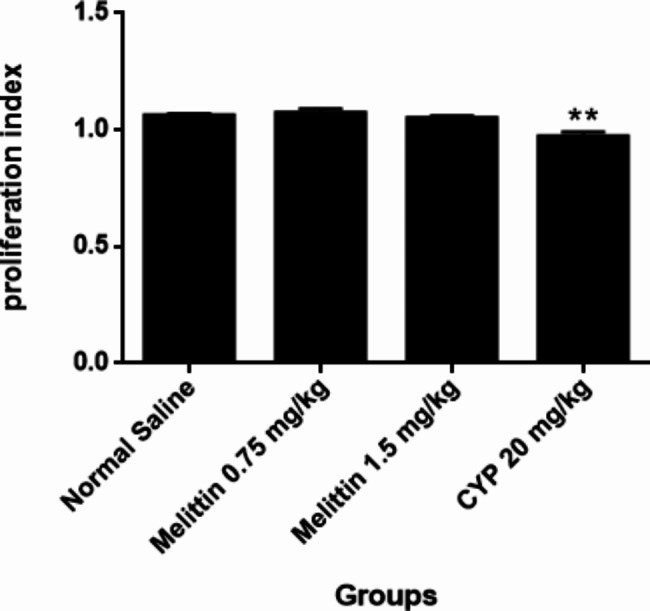
The subacute effects of i.p administration of melittin on LPS response in different groups of mice. Data are shown as mean ± SEM. **P < 0.01 indicates significant changes compared to the negative control group

Spleen cell viability in all groups of mice was found to be > 98%. Melittin did not induce any significant changes in splenocyte proliferation in the presence to PHA and LPS as compared to normal saline group. In other side, cyclophosphamide caused a meaningful reduction in proliferation indexes compared to the negative control group (P < 0.01) (Figs. 4 and 5).

### Hemagglutination titer assay

**Fig. 6:**
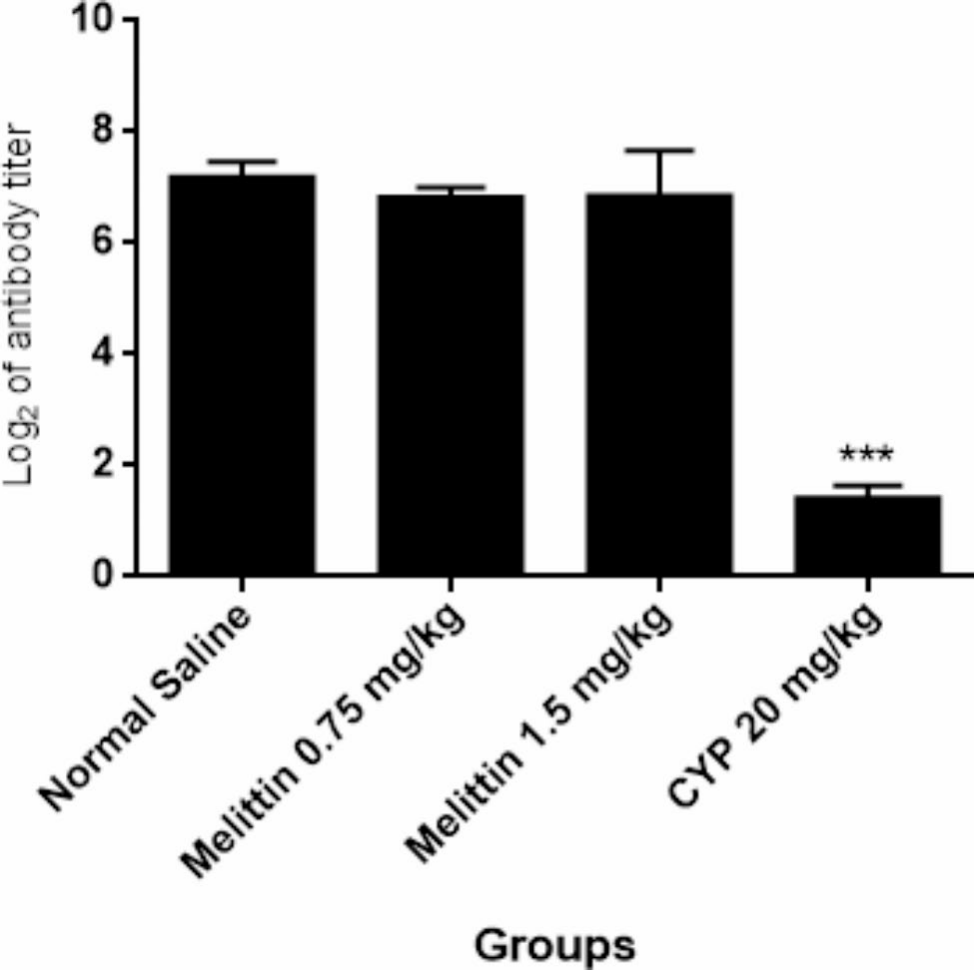
The subacute effects of i.p administration of melittin on the production of anti-SRBC antibodies in different groups of mice. Data showed as mean ± SEM. ***P < 0.001 indicates significant changes compared to the negative control group

Measures of serum anti-SRBC titer of 0.75 and 1.5 mg/kg melittin groups showed no significant changes relative to negative control group. On the other hand, cyclophosphamide as positive control significantly (p < 0.001) decreased the generation of anti-SRBC antibody as compared to normal saline group of mice (Fig. 6).

### Delayed-type hypersensitivity response (DTH)

**Fig. 7:**
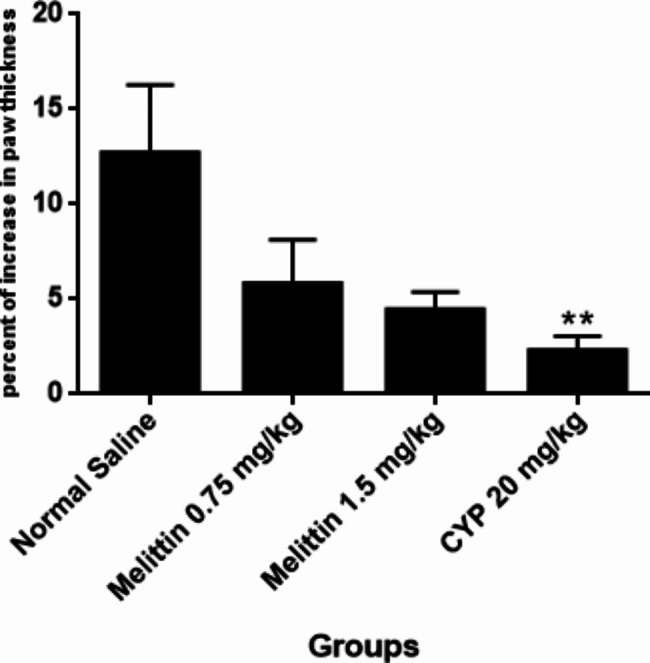
The subacute effects of i.p administration of melittin on 48 h-DTH response in different groups of mice. Data showed as mean ± SEM.**P < 0.01 indicates significant changes compared to the negative control group

In regard to evaluation of the 48 h-DTH response for melittin-treated groups, no statistical significant differences were observed in compared with the negative control, whereas the positive control group showed substantial suppression in DTH response (P < 0.01) (Fig. 7).

## Discussion

Natural compounds have been used for their beneficial effects for human health throughout the world. One of which is their use for the treatment of various ailments and diseases due to their limited side effects [22]. Honey bee venom is a great example of such compounds, and its main constituent is called melittin. A wide range of characteristics have been listed for melittin, as claimed by previous studies, such as antiviral, antimicrobial, and anticancer properties. According to high importance of immune system integrity in human body and in other side, having a limited number of studies regarding its effects on the immune system, we decided to investigate its effect on the BALB/c mice immune system based on a standard immunotoxicity screening protocols with a tiered approach.

Our results demonstrated that melittin at both administered doses (0.75 and 1.5 mg/kg), did not cause any significant changes in DTH, lymphocyte proliferation, cellularity of the spleen, splenic cell viability, HA, and hematological parameters. In addition, on histopathological point of view, melittin did not induce any meaningful changes in investigated histopathological indexes of the lymphatic organs (bone marrow and spleen) for the melittin treated groups of mice as compared to those of negative control animals.

Going through on the scientific databases, it turned out that several researches have been carried out in regard to melittin effects on different aspects of immune response for some diseases. For instance, in a study conducted by Lee et al., the effects of melittin on pulmonary and splenic inflammation observed in ALS (Amyotrophic lateral sclerosis) patients, were investigated [23]. In this study, melittin reduced the expression of inflammatory proteins such as CD14 and IBA-1 (ionized calcium-binding adaptor molecule-1) in the lungs, and CD14 along with COX2 in the mice spleen tissues. Therefore, it was concluded that melittin could be used as a regulatory agent for the immune system. It is worth mentioning that a dose of 0.1 mg/kg was administered in Lee et al. study which is lower than the doses used in our research (0.75 and 1.5 mg/kg). In another study, the anti-inflammatory effect of bee venom and specifically melittin on lipopolysaccharide (LPS)-stimulated BV2 microglia were evaluated [24]. Their results indicated that bee venom and melittin could suppress the transcription of COX2 genes and pro-inflammatory cytokines such as IL-1, IL-6, and TNF-α. As a result, melittin was recommended to be considered as a candidate for the treatment of neurodegenerative diseases. Alqarni et al. [25] performed a study in which the possibility of using melittin in adjuvant immunotherapy was evaluated. They did so by assessing whether or not melittin could enhance the release of cytokines from a macrophage cell line (THP-1) such as IL-1β, IL-6 and TNF-α when applied with or without LPS. Their findings suggested that the release of inflammatory cytokines induced by LPS was enhanced by the addition of melittin, and that melittin could potentially be used as a vaccine adjuvant. The aforementioned studies have been performed on humans with special diseases or macrophage cell line showing the relative immunoregulatory effects of melittin/bee venom. However, in the present study, we aimed to assess the immunotoxic/immunomodulatory effects of melittin in an immunotoxicity screening model of mice. All experimental tests used here were in consistent with immunotoxicity screening protocols for chemicals defined in known immunotoxicity guidelines [26].

Finally, with this approach, our findings demonstrated that melittin at the doses administered here, seems to be a safe compound with low toxicity. Of course, it is possible that melittin at doses higher than doses administered here may have side effects that outweigh its benefits/safety. Cytotoxicity is one of the aforementioned side effects that occurs when melittin incorporates itself into and disrupts phospholipid bilayers [27].

## Conclusion

In conclusion, despite several studies demonstrating the immunomodulatory effects for melittin, no significant alteration was made by this compound in the parameters of the immune system of mice at the administered doses (0.75 and 1.5 mg/kg) here. As a result, the current study exhibited that melittin was a safe compound for the immune system, and it could be recommended to be considered as a candidate for its antimicrobial and anticancer properties, especially in patients that suffer from autoimmune or immune deficiency diseases. On the other hand, conducting future studies with different doses could be considered to shed light on melittin’s immunomodulatory effects. In addition, further mechanistic studies with different doses of melittin are also recommended to be determined how it acts on immune system.

## Data Availability

The datasets used and analysed during the current study are available from the corresponding author on request.

## References

[1] Rahnama M, Mahmoudi M, Zamani Taghizadeh Rabe S, Balali-Mood M, Karimi G, Nafiseh Tabasi N (2015). Evaluation of anti-cancer and immunomodulatory effects of carnosol in a Balb/c WEHI-164 fibrosarcoma model. J Immunotoxicol.

[2] Mahmoudi M, Zamani Taghizadeh Rabe S, Balali-Mood M, Karimi G, Tabasi N, Riahi-Zanjani B (2015). Ursolic acid induced apoptotic cell death following activation of caspases in isolated human Melanoma cells. Cell Biol Int.

[3] Rybak M, Skubida P (2007). Application of coupled electrical and sound stimulation for honeybee venom collection. J Apic Sci.

[4] Sun L, Wang S, Tian F, Zhu H, Dai L (2022). Organisations of melittin peptides after spontaneous penetration into cell membrane. Byophys J.

[5] Zaiou M (2007). Multifunctional antimicrobial peptides: therapeutic targets in several human Diseases. J Mol Med.

[6] Bramwell VW, Somavarapu S, Outschoorn I, Alpar HO (2003). Adjuvant action of melittin following intranasal immunisation with Tetanus and Diphtheria toxoids. J Drug Target.

[7] Tichy J, Novak J (2000). Detection of antimicrobials in bee products with activity against viridans Streptococci. J Altern Complement Med.

[8] Lazarev VN, Shkarupeta MM, Titova GA, Kostrjukova ES, Akopian TA, Govorun VM (2005). Effect of induced expression of an antimicrobial peptide melittin on Chlamydia trachomatis and Mycoplasma hominis Infections in vivo. Biochem Biophys Res Commun.

[9] Yun SW, Bae GS, Kim MS, Park KC, Koo BS, Kim BJ (2011). Melittin inhibits cerulein-induced acute Pancreatitis via inhibition of the JNK pathway. Int Immunopharmacol.

[10] Carpena M, Nunez-Estevez B, Soria-Lopez A, Simal-Gandara J (2020). Bee venom: an updating review of its bioactive molecules and its health applications. Nutrients.

[11] Guha S, Ferrie RP, Ghimire J, Ventura CR, Wu E, Sun L et al. 2021. Applications and evolution of melittin, the quintessential membrane active peptide. Biochem Pharmacol. 2021;193:114769–816. 10.1016/j.bcp.2021.114769 PMC9235364 34543656

[12] Yu AR, Kim JJ, Park GS, Oh SM, Han CS, Lee MY (2012). The antifungal activity of bee venom against dermatophytes. J Appl Biol Chem.

[13] Hood JL, Jallouk AP, Campbell N, Ratner L, Wickline SA (2013). Cytolytic nanoparticles attenuate HIV-1 infectivity. Antivir Ther.

[14] Vento R, D’Alessandro N, Giuliano M, Lauricella M, Carabillò M, Tesoriere G (2000). Induction of apoptosis by arachidonic acid in human retinoblastoma Y79 cells: involvement of oxidative stress. Expe Eye Res.

[15] Putz T, Ramoner R, Gander H, Rahm A, Bartsch G, Thurnher M (2006). Antitumor action and immune activation through cooperation of bee venom secretory phospholipase A2 and phosphatidylinositol-(3, 4)-bisphosphate. Cancer Immunol Immunother.

[16] Son DJ, Lee JW, Lee YH, Song HS, Lee CK, Hong JT (2007). Therapeutic application of anti-arthritis, pain-releasing, and anti-cancer effects of bee venom and its constituent compounds. Pharmacol Ther.

[17] Jang MH, Shin MC, Lim S, Han SM, Park HJ, Shin I (2003). Bee venom induces apoptosis and inhibits expression of cyclooxygenase-2 mRNA in human Lung cancer cell line NCI-H1299. J Pharmacol Sci.

[18] Wang C, Chen T, Zhang N, Yang M, Li B, Lü X (2009). Melittin, a major component of bee venom, sensitizes human hepatocellular carcinoma cells to Tumor necrosis factor-related apoptosis-inducing ligand (TRAIL)-induced apoptosis by activating CaMKII-TAK1-JNK/p38 and inhibiting IκBα kinase-NFκB. J Biol Chem.

[19] Ip SW, Liao SS, Lin SY, Lin JP, Yang JS, Lin ML (2008). The role of mitochondria in bee venom-induced apoptosis in human Breast cancer MCF7 cells. In Vivo.

[20] Karimi G, Hassanzadeh-Josan S, Memar B, Esmaeili SA, Riahi-Zanjani B (2018). Immunomodulatory effects of silymarin after subacute exposure to mice: a tiered approach immunotoxicity screening. J Pharmacopunct.

[21] Vakili T, Iranshahi M, Arab H, Riahi B, Mohammadian Roshan M, Karimi G (2017). Safety evaluation of auraptene in rats in acute and subacute toxicity studies. Regul Toxicol Pharmacol.

[22] Danaei GH, Amali A, Karami M, Khorrami MB, RiahiZanjani B, Mahmood Sadeghi M (2022). The significance of thymoquinone administration on liver toxicity of diazinon and cholinesterase activity; a recommendation for prophylaxis among individuals at risk. BMC Complemnt Med Ther.

[23] Lee SH, Choi SM, Yang EJ (2014). Melittin ameliorates the inflammation of organs in an Amyotrophic Lateral Sclerosis animal model. Exp Neurobiol.

[24] Moon DO, Park SY, Lee KJ, Heo MS, Kim KC, Kim MO (2007). Y. Bee venom and melittin reduce proinflammatory mediators in lipopolysaccharide-stimulated BV2 microglia. Int Immunopharmacol.

[25] Alqarni AM, Ferro VA, Parkinson JA, Dufton MJ, Watson DG (2018). Effect of melittin on metabolomic profile and cytokine production in PMA-differentiated THP-1 cells. Vaccines.

[26] Pauels HG, Taylor J, Immunotoxicity Testing. ICH Guideline S8 and related aspects. Wiley Online Library; 2007.

[27] Lyu C, Fang F, Li B (2019). Anti-tumor effects of melittin and its potential applications in clinic. Curr Protein Pept Sci.

